# Comparison of in-person versus telephonic interview on tobacco cessation in an Indian dental setting

**DOI:** 10.6026/97320630019775

**Published:** 2023-06-30

**Authors:** Sathya Kumaresan, Sri Sakthi D, Arumugham Indiran Meignana, Arthi Balasubramaniam, Pradeep Kumar Rathinavelu

**Affiliations:** 1 Department Public Health Dentistry, Saveetha Dental college, Saveetha Institute of Medical and Technical Sciences, Saveetha University, Chennai, India

**Keywords:** Tobacco cessation, tobacco use cessation methods, telephone counseling.

## Abstract

Oral health professionals in the dental office settings have a distinctive opportunity to increase tobacco abstinence rates among tobacco users as tobacco use has significant adverse effects on oral health. This review assesses the effectiveness of tobacco cessation interventions offered to cigarette smokers and smokeless tobacco users in the dental office setting. The following electronic retrieval systems and databases were searched for the identification of studies, The Cochrane Central Register of Controlled Trials, PUBMED, GOOGLE SCHOLAR, SCIENCE DIRECT and TRIP. The review included randomized clinical trials assessing tobacco cessation interventions conducted by oral health professionals in the dental office setting. Seven clinical trials met the criteria for inclusion in this review. All the studies have employed behavioral therapy, telephonic counseling's and pharmacotherapy as interventional component. The rate of abstinence and biochemical validation were the outcome measurements. Since all the studies included were randomized clinical trials, the level of evidence was II. Available evidence suggests that telephonic interventions for tobacco use conducted by oral health professionals in the dental office setting may increase tobacco abstinence rates among smokers and smokeless tobacco users. This review data suggests that telephone has a pragmatic effect on interactional aspects of psychological therapy. Further research should be carried out to make conclusive recommendations regarding the intervention components that can be incorporated in the dental office settings.

## Background:

The global health impact of tobacco use is enormous [1]. More than 5 million deaths occur every year worldwide due to tobacco use based on the estimates of the World Health Organisation [2]. The bio-physical, psychological and social spheres of life are impaired by the ill effects of tobacco. Smoking is the predominant habit among males in India constituting more than 50% of the tobacco users. The prevalence of tobacco use among males in India is 48% compared with 20% among females according to the Global Adult Tobacco Survey [3]. Both behavioral and pharmacological support can improve motivation in smokers to quit. Behavioral approaches range from brief advice from a physician to intensive specialist counseling [4]. Many people who smoke do not wish to attend group programs, and the timing of group programs can also be inflexible. Individual counseling is more flexible but more expensive. Counselling sessions or group programmes [person-to-person contact] also impairs an increased loss in attendance and these are minimally prioritized [5]. Ease of use, whenever required, cost-effective delivery and scalability to a large number of people are numerous potential benefits of telephone counselling for smoking cessation [6], regardless of location. In addition, the ability to personally interact with the patients with exclusively curated content based on key characteristics like age , SES and tobacco dependence makes the intervention highly acceptable among the smokers [7]. It also helps in distracting the smokers from craving and also links the smokers with others for social support [8] [9]. Telephone contact can therefore maximize the level of support around a planned quit date, and can also be scheduled in response to the needs of the recipient. Indeed, World Health Organization identified mobile phone-delivered interventions as one of the most efficient and affordable interventions for global tobacco control. The rationale for this systematic review was, therefore, to establish what research evidence exists to support such claims about the effectiveness between telephone and face to face therapy. Therefore, it is of interest to present systematic review was to compare the effectiveness of in person interview and telephonic based interview on tobacco cessations in dental office settings. Table 1,Table 2,Table 3, Table 4

## Materials and Methods:

## Pico analysis:

Population: All tobacco users (cigarette, cigar, pipe smokers, smokeless tobacco users)

Intervention: Telephonic based interview

Comparison: In person interview

Outcome: Rate of abstinence and biochemical validation

Focused question: Is telephonic counselling non-inferior in tobacco use cessation in dental office settings over the conventional in person interview among tobacco users?

## Inclusion criteria:

[1] Studies considering telephone mode or featuring other empirical comparison with face to face.

[2] Randomized Clinical Trials

[3] All tobacco users (cigarette, cigar, pipe smokers, smokeless tobacco users)

[4] Details of participants, including whether they were selected according to motivation to quit, their age, gender and average baseline cigarette consumption.

[5] Description of intervention and control, including the number, timing, duration of telephone contacts.

[6] Use of biochemical validation

## Exclusion criteria:

· Not primary research(practitioner reflections, topic overviews, practice manuals/guides)

· Studies addressing something other than abstinence as the primary outcome of the study

· Studies published before the year 2000.

## Identification of studies:

The study followed the PRISMA guidelines for reporting systematic reviews and Meta analyses. To identify potentially relevant items, the following databases were searched: Pubmed, Cochrane Library, Google scholar, Science Direct and Trip.

## Data collection and analysis:

## Screening and selection:

**Figure 1:**
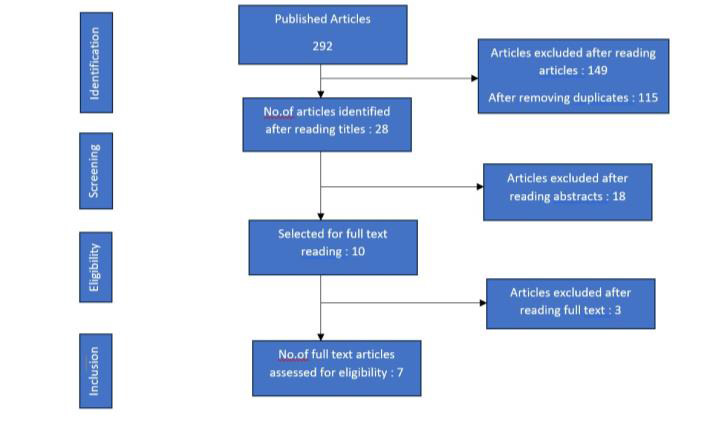
PRISMA flowchart for included studies

Electronic search was carried out using keywords in Search engines Pubmed, Science Direct, Cochrane, Google Scholar and Trip which yielded a total of 292 articles. Based on inclusion and exclusion criteria, the titles of studies identified from the search were assessed independently by three review authors (Dr. Sathya Kumaresan, Dr. Pradeep Kumar R and Dr. Arthi B). Conflicts concerning inclusion of the studies were resolved by discussion. One hundred and forty nine articles were identified from the search after reading the titles and selected for reading abstracts. Abstracts of the selected articles were reviewed independently. Eighteen studies were excluded after reading abstract. One hundred and fifteen studies were excluded for duplications. After reviewing the articles independently, finally seven articles were selected based on eligibility criteria. The reference list of full text articles were reviewed for identifying additional studies. Titles of articles relevant to review were selected by discussion. Quality Assessment criteria to evaluate the studies were decided by two review authors in accordance with CONSORT guidelines. The risk of bias for each study was independently assessed by the review authors and conflicts concerning risk of bias were sorted by discussion using Review Manager 5.3.(Figure 1)

##  Quality assessment: Cochrane risk of bias tool (Higgins 2011) -Review Manager 5.3.

**Figure 2:**
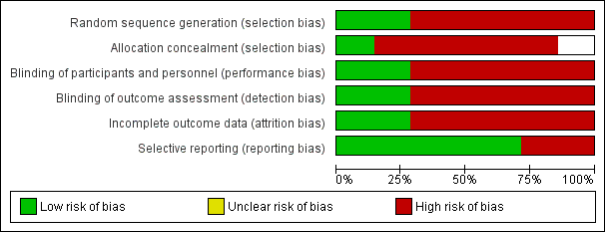
Risk of bias -Included Studies

**Figure 3:**
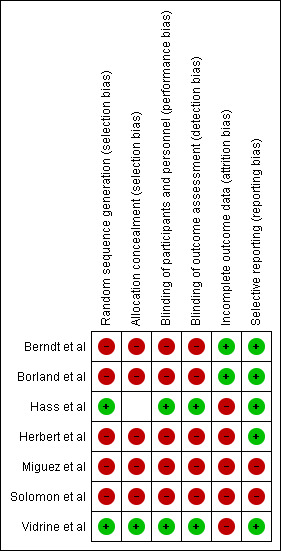
Risk of bias summary

The risk of bias assessment of the included studies used the approach recommended by Cochrane Collaboration's tool (Higgins *et al*. 2011). All the included studies were assessed independently by two review authors for study design characteristics and features of internal validity. Assessment was done within and across studies. The first step was writing a description of the results of each included study. Next involved was the assessment of risk of bias where a score of low, high or unclear was assigned for each included study. The overall quality of each study was then assessed by grading the six bias categories. A score of 3,1 and 0 were considered as low, unclear and high risk of bias respectively for each of the six categories of biases. Any disagreement was resolved by discussion or by third party adjudication (Figure 2 and Figure 3).

The AHRQ classifies the studies in seven levels according to the level of evidence

[1] Systematic review or Meta - analysis

[2] Randomized controlled trials

[3] Controlled trials without randomization

[4] Case control and Cohort studies.

[5] Systematic review of descriptive and qualitative studies

[6] Single descriptive or qualitative study

[7] Opinion of authorities and or report of expert committees.

## Results:

None of the studies described the method of randomization in sufficient to exclude the possibility of allocation bias. In one of the trials, low socioeconomic status adult smokers recruited by Interactive Voice Response were the units of randomization. Similarly, in another study, socioeconomically disadvantaged individuals recruited from churches, public housing complexes and community centers were the units of randomization. In a study by Herbert H, military personnel from 24 military dental clinics across the U.S were randomized. Likewise, in a study conducted by Ma Carmen, advertisements in newspapers, radio and local TV were used to randomize tobacco users. Furthermore, in a trial conducted by Laura J Solomon, Medicaid eligible women smokers of child bearing age were randomized. In one study, the participants were given access to a hotline according to the country of residence so that the availability of hotline could be advertised in the intervention countries. In a study by N. Berndt, patients admitted to cardiac wards were randomized.

In seven trials, biochemical validation was done in one study conducted by Damon Vidrine. Biochemical validation estimated the amount of salivary cotinine levels. Many trials reported sustained abstinence at one or more follow-ups. One trial reported with a short-term point prevalence of 7-day abstinence after a 9 month follow up. Long term sustained abstinence or abstinence at one or more previous follow-ups is used as the outcome for almost all trials. Length of longest follow up ranged to 12 months after the end of intervention.

## Telephone counseling and behavioral treatment versus telephone based motivational counseling and NRT:

There were two trials included in this category. In the study based on motivational counseling and NRT, the IVR sends an automated e-mail to the tobacco treatment specialists on request. The session comprised of 4 counseling calls approximately from 75 to 100 min for all calls over 8-10 weeks. The counseling calls were content tailored based on the intent and confidence to quit. Supply of NRT patches were based on the consumption of cigarettes. Participants who smoked 10 or more cigarettes per day were offered 2 weeks supply of 21 mg/d patches, 2 weeks supply of 14mg/d patches,2 weeks supply of 7mg/d patches. Participants who smoked lesser than 10 cigarettes per day were offered 4 weeks supply of 14 mg/d patches, 2 weeks supply of 7mg/d patches. The intervention group reported with a higher quitting rate. Likewise, the intervention for behavioral treatment included smokeless tobacco cessation manual, videotape cessation guide, three sessions of 15 min telephone counseling. The mean duration of call 1 is 17.3 min, call 2 is 18.4 min and call 3 is 14.1 min. The participants in the telephone intervention group were more likely to be abstinent for 6 months at 16.8% quit rate as compared to 6.4% of the usual care group.

## Standard versus Telephone Counseling alone:

Two studies fall under this category. The first study offers a biochemical validation. It measures the amount of carbon monoxide expelled in air. Here the intervention group received 6 calls, of which in the first 4 calls, the counselor provided motivational and cessation strategies and the last 2 were maintenance strategies. In the second study, no biochemical validation was assessed.3 month follow up shows 24% quit rate in the intervention group while 13% in the usual care group.

##  Discussion:

Low socioeconomic status (SES) smokers have a more difficulty quitting for several reasons, including a limited access to treatment and lack of social support. In addition, there also exists a misreport about risks and benefits of nicotine replacement therapy (NRT).Systematic telephonic intervention facilitated by oral health professionals may be significantly important for low-SES smokers who experience substantial barriers to tobacco treatment The review reveals that available evidence is accordant with the hypothesis that telephone based counselling interventions conducted in the dental office can be more effective than usual care for tobacco cessation. These conceptual models of smoking cessation allow the possibility of large scale linkage to provide support for tobacco treatment. Coupled with telephones or other technology, this infrastructure stresses the importance of addressing the ill effects of smoking on a broader context.

The study conducted by Jennifer S. Haas has incorporated Interactive Voice Response (IVR) in the phone technology [10]. IVR has been used as part of multi-component smoking cessation programs to provide reminders and facilitate or sustain treatment delivery. The counselling calls included standard content, as well as tailored content for the individual based on intent and confidence to quit. Among individuals in the intervention arm, individuals who spoke to the TTS (Tobacco Treatment Specialist) were more likely to quit compared to those who did not (21.2% vs. 10.4%, p=0.009). In the study conducted by Damon, higher cessation rates among participants were observed in tailored interactive text messages and proactive counselling via mobile phone compared to a lower intensity control group. The results of the study suggest that offering low intensity treatment such as nicotine patches and referral to quit line services may be an effective approach in treating smokers. In this randomized clinical trial, biochemically verified abstinence was found 19 (12.0%) with the addition of text messaging, and 28 (25.5%) with the addition of text messaging plus call [11]. Findings from a similar trial conducted for smokers in Denmark stated that participants who received proactive counselling were more likely to quit smoking successfully in comparison with participants who received self-help materials [12].

The results of the study conducted by Herbert showed that military personnel who received a telephone-based behavioural intervention were more likely to quit all tobacco use. The findings suggest that the no. of phone calls completed was positively related to outcomes but negatively related to the length of phone calls [13]. These finding are consistent with the fact that phone calls are an integral component of the tobacco intervention. The study conducted by Ma. Carmen Miguez showed that multiple counselling calls before the quit date facilitates not only in reducing the relapse, but also increasing the rate of abstinence as well. In addition, the results suggest that the effects of the telephone based tobacco intervention may be generalized to other countries [14]. The study conducted by Laura Solomon suggested that gradual cessation was not superior to abrupt cessation nor minimal treatment treatments among smokers who preferred to quit naturally [15]. However, a post-hoc finding suggested that a gradual cessation might be equivalent to abrupt cessation in more dependent smokers. A "reduce-to-quit" indication for NRT has been approved in several countries for smokers who plan to quit [16]. Reduction consistently increases the probability of later quit attempts and abstinence among such smokers [17]. Thus, the study concludes that reduction is efficacious in unsure smokers who do not plan to quit in the near future than in motivated smokers who want to quit soon.

According to Ron Borland, GP_s_ have a responsibility to indicate the harms associated with smoking to their patients who smoke and encourage and support them to quit whenever needed. Brief counselling from a GP to as short as 3 min can substantially increase quit rates. Motivation to quit is the predominant effect of counselling. These findings report that GP_s_ referring smokers to quitline service receive more external help than patients in the in-practice condition that results in an increased cessation [18]. According to N. Berndt, short-term cost savings and greater smoking abstinence rates are enhanced by multiple telephone and face-to-face-delivered counselling interventions than usual care [19]. A previous study conducted for the general population of Dutch smokers also revealed a higher cost-effectiveness for counselling delivered by telephone than for counselling delivered face-to-face [20].

## Conclusion:

The telephone mode of anti-tobacco counseling does evidently make a difference amongst the tobacco cessations. However, effecting a change in practice mandates more than simply informing practitioners of this scientific based evidence. To bring about a change in the patients attitude and behaviors, more prospective forms of interventions are required. The barriers to change not only lie at the individual but also at the system level. As the main component of an intervention, proactive telephone counseling helps smokers to quit. It is apparent that telephone quit lines provide a principal access of support to smokers and that a call from a counselor is likely to increase the chances of quitting relatively by around 50%.To conclude, telephone counselling serves as an effective aid in smoking cessation program in dental office settings. However, more research is needed to clarify the underlying therapeutic mechanisms like the optimal number and length of telephone contacts needed for an enhanced cost-effectiveness of the program.

## Figures and Tables

**Table 1:** variables of interest

**Sr. No.**	**Variables of interest Self-reported 30 day abstinence**
1	Prolonged Abstinence rate at 6 months
2	Biochemically verified abstinence-salivary cotinine

**Table 2:** Level of Evidence according to Agency for Health Research and Quality (AHRQ) guidelines (2016)

**Sr. No.**	**Author and Year**	**Study Design**	**Level of Evidence**
1	Jennifer S. Haas, 2015	Clinical Trial	II
2	Damon Vidrine, 2018	Clinical Trial	II
3	Herbert H, 2009	Clinical Trial	II
4	Ma Carmen Miguez, 2002	Clinical Trial	I1
5	Laura Solomon, 2010	Clinical Trial	II
6	Ron Borland, 2008	Clinical Trial	II
7	N. Berndt, 2016	Clinical Trial	II

**Table 3:** Data extraction

**S.no**	**Article**	**AuthorandJournal**	**Samplesize**	**Methodology**	**Interventions**	**Control**	**StatisticalAnalysis and Results**
1	Proactive tobacco cessation outreach to smokers of low socioeconomic status A Randomized clinical trial	Jennifer.S.Haas, 2015. JAMA Internal Medicine 2015;175(2);218-226	Sample size N= 702 of which N=399 (Intervention group) N=303 (control group) Mean age=50 years, Females=68%, 20% Hispanic,28% black	This prospective randomized clinical trial included low SES adult smokers who described their race and or ethnicity as Black, Hispanic or White and received primary care at 13 practices in Greater Boston	1.Telephone based motivational counselling 2.Free NRT for 6 weeks 3.Access to community-based referrals to address socio contextual mediators of tobacco use	Usual care	Subgroup Analyses: Individuals who participated in telephone counselling were more likely to quit than those who did not (21.2% vs 10.4%).
2	Efficacy of mobile phone delivered smoking cessation interventions for socioeconomically disadvantaged individuals. A randomized clinical trial.	Damon Vidrine, 2018. JAMA Internal Medicine 2019;179(2):167- 74	Sample size N=624 of which N=223(NRT) N=213(NRT plus Text) N=188(NRT plus Text plus Call) Females=50.6%, mean age=45.8 years Average of 5 cigarettes per day	A group randomized clinical trial with neighbourhood site serving as the sampling unit	Group 1:NRT plus Text messages, mobile phone text message Group 2: NRT NRT plus Text messages plus proactive counselling via mobile phone calls	NRT- Transdermal nicotine patches.	Generalized linear mixed model analyses: Abstinence rate was 12% for NRT, 12% for NRT plus text message, 25.5% for NRT plus Text messages plus proactive counselling via mobile phone calls
3	Effectiveness of telephone contact as an adjunct to a self-help program for smoking cessation: A randomized controlled trial in Spanish smokers	Ma.Carmen Miguez, 2002. Addictive BehaviorsVol 27, Issue 1,2002.	Sample size N=200 of which N=100 (Standard self-help group) N=100 (Self-help group receiving additional telephone counselling) Average number of 27 cigarettes per day	The participants were randomized into two study groups. At the 12th month follow up, the carbon monoxide in expired air was used to distinguish smokers from non-smokers,	Telephone counselling group received 6 calls (each one week apart) Each call lasted for 10 mins.	Self-help group 1: Personalized letter assigning weekly reading and tasks 2 : Seven self-monitoring forms 3 : Self evaluated adherence form	Pairwise comparison between the two groups. Continuous abstinence rate at 3 months follow up was 21% and 48% for self-help group and self-help plus telephone counselling group.
4	Smokeless tobacco cessation in military personnel:A randomized controlled trial	Herbert H.2009 Nicotine and Tobacco Research Volume 11, 2009.	Sample size N=785 of which N=392(Minimal contact behavioural treatment) N=393(Usual care)	Participants were recruited from 24 military dental clinics across the United States during dental examinations.	Behavioural treatment included 1.Cessation manual 2.videotape 3. Three telephone counselling of 15 min.		Multiple logistic Regression Abstinence rate for Behavioural treatment was 16.8% and for usual care was 7.6% in 6 months follow up.
5	A randomized controlled trial of NRT aided gradual versus abrupt cessation in smokers actively trying to quit	Laura Solomon,2010 Drug and Alcohol Dependence Volume 111, Issue 1, 2010.	Sample size N=746 of which N=297(Gradual cessation) N=299(Abrupt cessation) N=150 (Minimal treatment)	Participants were recruited via newspaper and radio advertisements. Primary outcome-prolonged abstinence-post quit day.	Gradual and abrupt conditions - 5 phone calls-90 min. Minimal treatment condition – 2 calls – 25 min. After the quit day, all participants received lozenges.	-	Fewer smokers in the gradual condition (48%) made a quit attempt than in the abrupt (64%) or minimal (60%) conditions
6	In practice management versus quilting referral for enhancing smoking cessation in general practice - A Cluster Randomized trial	Ron Borland, 2008 Family Practice Volume 25, Issue 5, 2008.	Sample size N= 998 of which N=497(Callback counselling) N=501(Usual care)	Practices were randomized to one of two interventions at a ratio of 1:2. Main outcome measures were sustained abstinence of >1 month duration at 3 month follow up and >10 months duration at 12 months	Standard in practice GP management	Referral to quitline service	Mediated Regression Analysis. At 12 month follow up, patients in the referral condition had nearly three times the odds of sustained abstinence (6.5% compared with 2.6%, OR = 2.86.
7	Economic evaluation of telephone and face to face delivered counselling intervention for smoking cessation in patients with coronary heart disease	N.Berndt, 2016 The European Journal of Health Economics 17,269-285 (2016)	Coronary heart disease patients: N=245(usual care) N=223(Telephone counselling) N=157(Face to face counselling)	The counselling interventions lasted for 3 months and were complemented by nicotine patches.	Group 1: Each telephone counselling comprised of 15 min approx.	Usual care	Sensitivity Analysis.

**Table 4:** Summation table for outcome:

Author	Year	Evaluation Period	Outcome	Inference	Level of Evidence	Limitations
Jennifer S.Haas	2015	Self-reported-7 day tobacco abstinence at 9 months	Abstinence and automated caller validation	Individuals who participated in telephone counseling were more likely to quit than those who did not (21.2% vs 10.4%).	Low	Imbalanced group sizes, Pragmatic trials
Damon Vidrine	2018	6 months	Validation-Biochemical, salivary cotinine level and self-reported 30 day abstinence	Abstinence rate was 12% for NRT, 12% for NRT plus text message, 25.5% for NRT plus Text messages plus proactive counseling via mobile phone calls	Moderate	Factorial trial would be necessary to evaluate the treatment element of two groups
Herbert H	2009	3 months and 6 months follow up	Repeated point prevalence at 3 months and 6 months	Abstinence rate for Behavioral treatment included Cessation manual, videotape, three telephone counseling of 15 min was 16.8% and for usual care was 7.6% in 6 months follow up.	Low	Study is unable to determine related cost effectiveness of behavioral intervention
Ma. Carmen Miguez	2002	3 months and 6 months follow up	Repeated point prevalence at 3 months and 6 months	Continuous abstinence rate at 3 months follow up was 21% and 48% for self-help group and self-help plus telephone counseling group Continuous abstinence rate at 6 months follow up was 18% and 40% for self-help group and self-help plus telephone counseling group	Moderate	Smoking cessation rate based on self-help report need to be interpreted conservatively.
Laura Solomon	2010	Baseline- 10 days, 3 months and 6 months	Repeated point prevalence at 3 months and 6 months	Abstinence rate for NRT 28% and NRT + proactive telephone call was 42% at 3 months follow up.	Low	Relapse rate was high in the experimental group between 3 and 6 month follow up
Ron Borland	2008	3 and 6 months	3 months follow up with 24% quit rate of call back counseling and 13 % quit rate in usual group	3 months follow up abstinence rate for callback group was 24% and usual care was 13%.	Moderate	Timing of telephone calls before cessation and round relapse sensitive times after quitting leads to bias
N. Berndt	2016	12 month follow up	Abstinence and automated caller validation	Both the telephone and face to face counseling interventions was cost effective than the usual care. The intention to quit was 7.54% for usual care, 7.55% for telephone counseling and 2.06% for face to face counseling.	Moderate	Selective sample of cardiac patients limits the generalizability of the study.
